# Effect of axial length on peripapillary microvasculature: An optical coherence tomography angiography study

**DOI:** 10.1371/journal.pone.0258479

**Published:** 2021-10-14

**Authors:** Kee Sup Park, Hyung Bin Lim, Yong Il Shin, Gi Seok Park, Woo Hyuk Lee, Jung-Yeul Kim

**Affiliations:** 1 Department of Ophthalmology, Chungnam National University College of Medicine, Daejeon, Republic of Korea; 2 Department of Ophthalmology, Konyang University College of Medicine, Daejeon, Republic of Korea; 3 Myung Eye Clinic, Daejeon, Republic of Korea; 4 Rhee’s Eye Hospital, Daejeon, Republic of Korea; 5 Department of Ophthalmology, Gyeongsang University College of Medicine Changwon Hospital, Changwon, Korea; Mayo Clinic Scottsdale: Mayo Clinic Arizona, UNITED STATES

## Abstract

**Objective:**

To investigate the effects of axial length (AL) on the peripapillary microvascular density acquired from optical coherence tomography angiography (OCTA).

**Methods:**

Retrospective observational study. A total of 111 eyes from 111 normal healthy subjects were examined. The subjects were divided into three groups according to the AL: Group 1 (AL: < 24.0 mm; 35 eyes), Group 2 (AL: 24.0–25.99 mm; 37 eyes), and Group 3 (AL: ≥ 26 mm; 39 eyes). Peripapillary OCTA images were acquired using 6× 6 mm angiography scans, and vessel density (VD) and perfusion density (PD) of the superficial capillary plexus were calculated automatically. VD and PD were compared among the three groups according to the distance from the optic disc (inner and outer rings). Linear regression analyses were also performed to identify clinical factors associated with average VD.

**Results:**

The average ALs of Groups 1–3 were 23.33± 0.57, 25.05± 0.60, and 27.42± 0.82, respectively. Average VD (P = 0.009) and PD (P = 0.029) in the inner ring increased with increasing AL. However, average VD (P < 0.001) and PD (P < 0.001) in the outer ring decreased with AL increased; the same trends were found for the full areas (VD, p<0.001; PD, p = 0.001). Average VDs in the inner and outer rings were not associated (P = 0.938).

**Conclusions:**

Peripapillary VD and PD were significantly associated with AL. Depending on the distance from the disc, peripapillary VDs and PDs of the inner and outer rings were differentially affected by AL. Physicians should therefore consider the effects of AL in the analyses of peripapillary microvasculature.

## Introduction

Myopia is one of the most common ophthalmic diseases and a major cause of decreased visual acuity. However, the cause of myopia and its prevention are not well-known [1, 2]. Myopia prevalence varies according to region and race. The prevalence in east Asia, such as in China, Japan, and Singapore, is increasing, and the prevalence is also high among Jews [1]. High myopia can cause severe vision loss resulting from various ophthalmic diseases such as glaucoma, cataracts, retinal detachment, and myopic macular degeneration [3–6].

Optical coherence tomography (OCT) is a noninvasive method that can be used to obtain high-resolution cross-sectional images of multiple layers of the retina. OCT angiography (OCTA) is a new and noninvasive imaging modality that allows microvascular visualization of the retina and choroid in various layers, as well as quantitative measurement of perfusion, including in the optic nerve head, peripapillary, and macular areas [7–9].

Previous studies have reported changes in OCT and OCTA measurements according to axial length (AL) [10–13]. It was reported that the thickness of the retinal nerve fiber layer (RNFL) decreased in OCT measurements with longer ALs. In one study, OCTA measurements of vessel density (VD) were higher with increasing AL in the central and peripheral macula [14]. Nonetheless, there is still a lack of studies concerning the effect of AL on blood flow around the optic disc. The purpose of this study was to evaluate possible correlations between AL and measurements of VD and perfusion density (PD) in peripapillary areas and to examine parameters affecting the measurements of VD and PD.

## Methods

This study adhered to the tenets of the Declaration of Helsinki, and the study protocol was approved by the institutional review board of Chungnam National University Hospital in the Republic of Korea. The requirement for obtaining informed patient consent was waived due to the retrospective nature of the study by Institutional Review Board.

### Participants

We retrospectively analyzed patients who visited Chungnam National University Hospital from June 2017 to August 2018. Patients with normal eyes who had no history of eye disease or ocular surgery were included in the study. The exclusion criteria were: 1) a medical history of diabetes, hypertension, and any kind of ophthalmic disease that could affect peripapillary RNFL thickness, such as glaucoma, retinal diseases, and neuro-ophthalmic diseases; 2) best-corrected visual acuity (BCVA) < 20/25 or intraocular pressure (IOP) > 21 mmHg; or 3) any abnormal findings from slit lamp microscopy and fundus examination. Among the subjects who first visited our clinic for various reasons (health screening checkup, routine check for ocular disease such as cataract, peripheral vitreous floater, etc), those who met inclusion and exclusion criteria enrolled. If both eyes met the eligible criteria, one eye was randomly selected. All patients underwent a basic ophthalmic examination, including measurements of BCVA using a Snellen chart, IOP, the spherical equivalent (SE), and AL using an IOL Master (Carl Zeiss, Jena, Germany); slit-lamp biomicroscopy; a dilated fundus examination; spectral-domain OCT (SD-OCT); and OCTA. The subjects included in the study were divided into three groups according to AL: Group 1 subjects had AL < 24 mm, Group 2 subjects had AL 24.0–25.99 mm, and Group 3 subjects had AL ≥ 26 mm.

### OCT and OCTA measurement

SD-OCT imaging was performed by an experienced examiner using a Cirrus HD-OCT (Carl Zeiss Meditec, Dublin, CA, USA). An optic disc cube 200 × 200 scan mode was used for RNFL measurements, and the average RNFL thickness was analyzed. OCT images with signal strength (SS) < 7, poor centration, or segmentation error were excluded.

**Fig 1:**
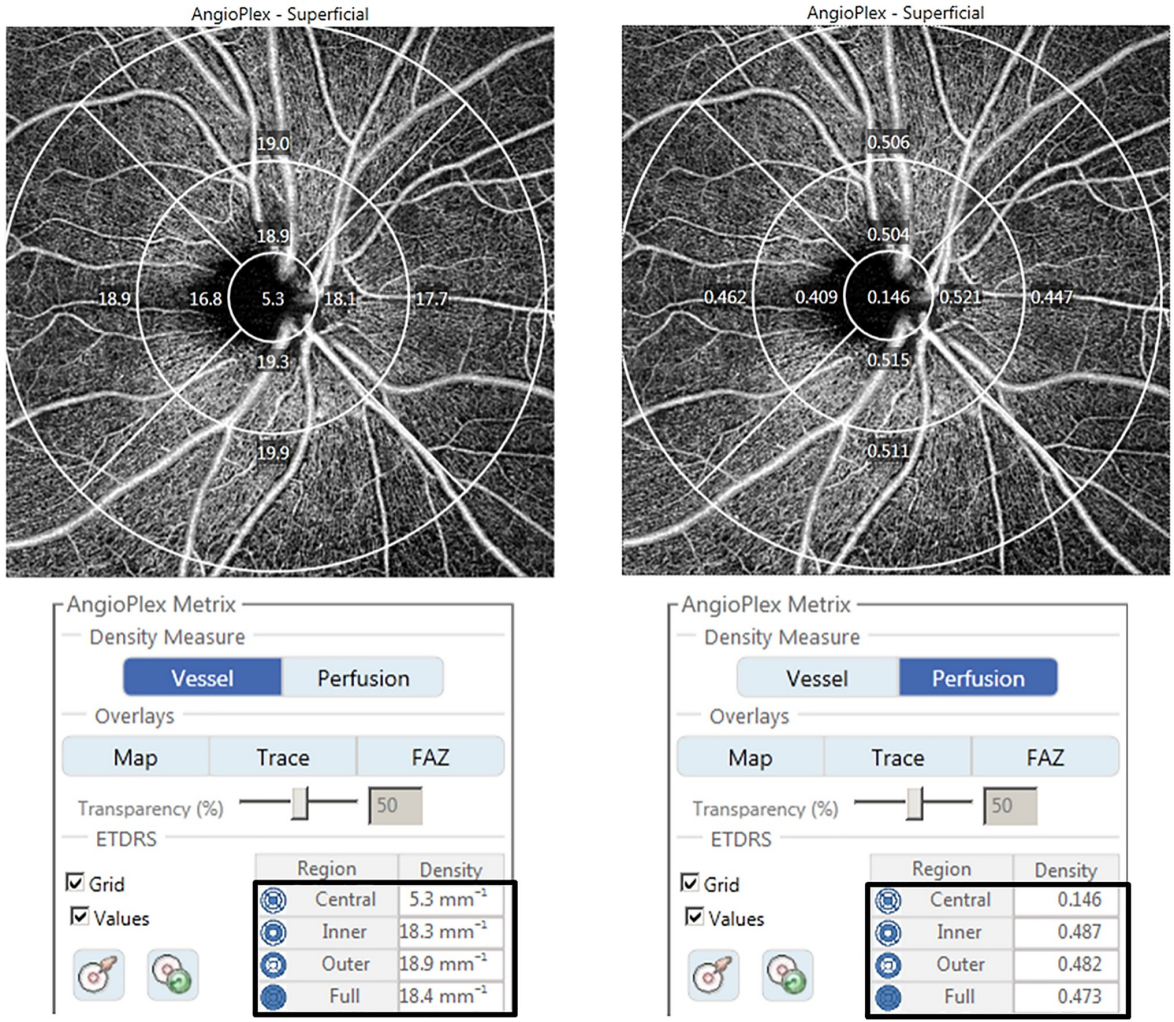
A 6 x 6-mm optical coherence tomography angiography image centered on the optic disc. The en face image of the superficial layer was overlaid with the Early Treatment of Diabetic Retinopathy Study grid. The diameters of the three concentric circles are 1, 3, and 6 mm. Using AngioPlex software (ver. 10.0; Carl Zeiss Meditec), we measured peripapillary (A) vessel density (VD) and (B) perfusion density (PD) in the individual subfields. Tables display the automatic measurements for density means in the central area, inner ring, outer ring, and full area.

OCTA images were obtained by a single experienced examiner using a Cirrus HD-OCT 5000 with an AngioPlex device (Carl Zeiss Meditec) and using a wavelength of 840 nm and an A-scan rate of 68,000 scans/s. To investigate peripapillary microvasculature, OCTA was performed using a 6 × 6 mm scan centered on the optic disc. In the 6 × 6 mm scan pattern, there were 350 A-scans in each B-scan along the horizontal dimension, and 350 B-scans were repeated twice at each location. The vascular images of the superficial capillary plexus (SCP), which spanned from the internal limiting membrane to the inner plexiform layer, and the deep capillary plexus, which extended from the inner nuclear layer to the outer plexiform layer, were displayed separately. VD, the total length of perfused vasculature per unit area in a region of measurement, and PD, the total area of perfused vasculature per unit area in a region of measurement, in the SCP were defined according to the Early Treatment of Diabetic Retinopathy Study subfields and were automatically measured using AngioPlex software (ver. 10.0). We analyzed the peripapillary VD and PD of the SCP in quadrants of the inner and outer rings, and calculated average values for inner ring, outer ring, and full areas (Fig 1). The diameters of the three concentric circles in 6 × 6 mm scans were 1, 3, and 6 mm, respectively, and each ring (inner and outer rings) was divided into four quadrants (superior, nasal, inferior, and temporal). OCTA images with SS ≥ 9 and those without motion artifacts and segmentation errors were included in the analysis.

**Fig 2:**
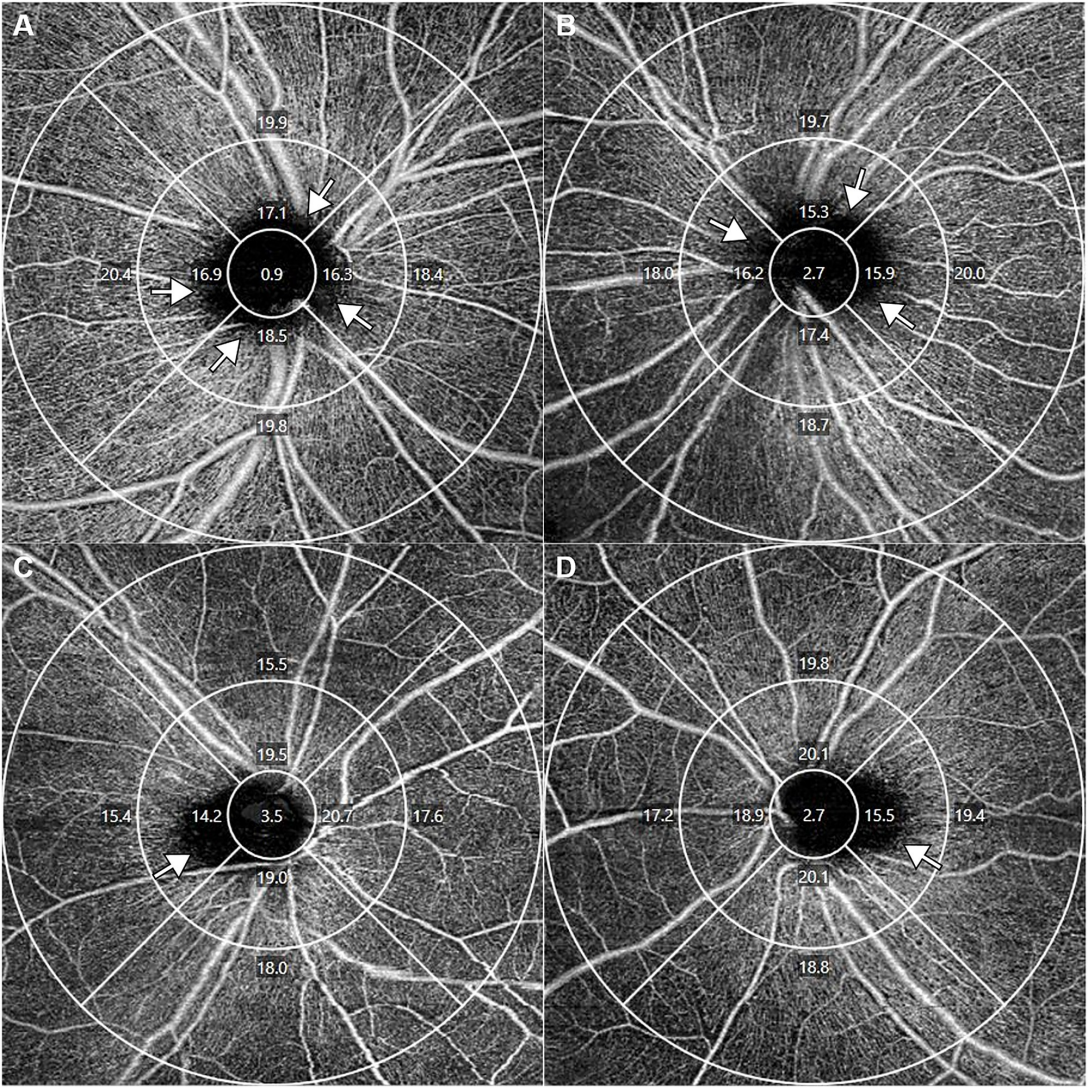
Representative optical coherence tomography angiography images of Group 1 (A and B, axial lengths of 22.14 mm and 22.67 mm) and Group 3 (C and D, axial lengths of 29.76 mm and 28.74 mm). The area where peripapillary vessels were not detected was widely observed throughout the inner ring in Group 1 (arrow), but only in the temporal sector in Group 3.

In addition, we also found that image artifacts occur in the peripapillary area in the OCTA images due to disc structural problem (such as disc tilting). Therefore, we performed additional analysis to find how the image artifacts (we defined this as “Black area”, Fig 2) according to the AL elongation affects the OCTA images using FIJI software (an expanded version of ImageJ version 1.51a, available at fiji.sc, free of charge).

### Statistical analyses

Statistical analyses were performed using SPSS statistical software for Windows (ver. 22.0; IBM Corporation, Armonk, NY, USA). The primary endpoint of this study was to determine the effects of AL on the OCTA measurements in peripapillary areas. Secondary endpoint was to find the factors other than AL (such as age, sex, BCVA, IOP, spherical equivalent, average RNFL thickness, average VDs, and image artifacts) that significantly affect the OCTA measurements. One-way analysis of variance (ANOVA) and chi-square tests were used to compare patient characteristics among groups. One-way ANOVA with Bonferroni correction was used to compare RNFL thickness, VD, and PD among the three groups. In addition, we compared OCTA measurements among the three groups adjusting for clinical variables using analysis of covariance (ANCOVA). A linear regression analysis was performed to analyze the relationships between OCTA measurements for the inner and outer rings and various clinical factors, such as age, sex, BCVA, spherical equivalent, IOP, AL, average RNFL thickness, and average VDs. For statistical analyses, BCVA values were transformed to the logarithm of the minimum angle of resolution (log MAR). A value of P < 0.05 was considered statistically significant.

## Results

### Patient demographics

A total 128 subjects were initially included in this study; 17 individuals were excluded due to poor image quality (low signal strength or segmentation error). As a result, 111 eyes of 111 patients were included in the study. These included 35 eyes in Group 1, 37 eyes in Group 2, and 39 eyes in Group 3. The average ages of patients in each group were 46.77 ± 11.62, 42.68 ± 12.64, and 41.77 ± 9.56 years, respectively (P = 0.139). ALs were 23.33 ± 0.57, 25.05 ± 0.60, and 27.42 ± 0.82 mm (P < 0.001), and spherical equivalents were –0.44 ± 1.33, –1.50 ± 1.84, and –5.21 ± 3.63 diopters, respectively (P < 0.001). Average RNFL thicknesses were 98.29 ± 9.50, 96.70 ± 10.97, and 87.67 ± 11.32 μm, respectively; these decreased significantly with increasing AL (P < 0.001). Average age, sex, BCVA, IOP, and signal strength did not differ significantly among the three groups (Table 1).

**Table 1:** Demographics and baseline patient characteristics.

	Group 1	Group 2	Group 3	p-value
	(< 23.99 mm)	(24–25.99 mm)	(≥ 26 mm)	
Eyes (No.)	35	37	39	
Age (years)	46.77 ± 11.62	42.68 ± 12.64	41.77 ± 9.56	0.139*
Sex (male:female)	13:22	24:13	18:21	0.055†
BCVA (logMAR)	0.04 ± 0.08	0.05 ± 0.07	0.04 ± 0.07	0.659*
Spherical equivalent (diopters)	-0.44 ± 1.33	-1.50 ± 1.84	-5.21 ± 3.63	**< 0.001** *
IOP (mmHg)	14.60 ± 2.56	14.81 ± 2.69	14.15 ± 3.40	0.606*
Axial length (mm)	23.33 ± 0.57	25.05 ± 0.60	27.42 ± 0.82	**< 0.001** *
Signal strength	9.63±0.47	9.73±0.46	9.48±-0.39	0.103*
Average RNFL thickness (μm)	98.29 ± 9.50	96.70 ± 10.97	87.69 ± 11.32	**< 0.001** *

Values are presented as means ± standard deviations.

BCVA, best corrected visual acuity; IOP, intraocular pressure; RNFL, retinal nerve fiber layer; logMAR, logarithm of the minimum angle of resolution.

*P-value from one-way analysis of variance.

^†^P-value from the chi-squared test.

Significant P-values are bolded.

### Comparisons of OCTA parameters according to axial length

The VD of the full area decreased significantly with increasing AL, with average values of 18.61 ± 0.55, 18.42 ± 0.66, and 17.78 ± 1.23 mm-1 for Groups 1–3, respectively (P < 0.001, Table 2). Average PDs exhibited a similar trend, with values of 0.47 ± 0.01, 0.47 ± 0.02, and 0.45 ± 0.03 for Groups 1–3, respectively (P = 0.001). Post hoc analysis revealed that the VDs and PDs of Group 3 were significantly lower than those of Groups 1 and 2 (all P < 0.01).

**Table 2:** Comparison of vessel density and perfusion density among three groups categorized according to axial length.

			Group 1	Group 2	Group 3	P-value*	P-value†	P-value‡	P-value§
			(< 23.99 mm)	(24–25.99 mm)	(≥ 26 mm)				
VD (mm-1)		6 mm Full area	18.61 ± 0.55	18.42 ± 0.66	17.78 ± 1.23	**< 0.001**	1.000	**<0.001**	**0.005**
	Inner ring	Average	17.83 ± 0.93	18.24 ± 0.96	18.58 ± 1.17	**0.009**	0.266	**0.006**	0.465
		Superior	18.12 ± 0.98	18.62 ± 1.02	19.00 ± 1.37	**0.006**	0.206	**0.004**	0.457
		Nasal	17.82 ± 1.61	18.25 ± 1.46	18.48 ± 1.21	0.135	0.601	0.145	1.000
		Inferior	18.09 ± 0.81	18.35 ± 0.90	18.94 ± 1.26	**0.002**	0.812	**0.001**	**0.040**
		Temporal	17.19 ± 1.64	17.60 ± 1.40	17.92 ± 2.20	0.215	0.975	0.242	1.000
	Outer ring	Average	19.43 ± 0.60	19.06 ± 0.81	18.00 ± 1.43	**< 0.001**	0.366	**< 0.001**	**< 0.001**
		Superior	19.42 ± 0.74	19.31 ± 0.61	18.57 ± 1.55	**0.001**	1.000	**0.003**	**0.009**
		Nasal	18.59 ± 1.00	18.17 ± 1.30	16.54 ± 1.83	**< 0.001**	0.653	**< 0.001**	**< 0.001**
		Inferior	19.70 ± 0.66	19.09 ± 1.06	17.83 ± 1.94	**< 0.001**	0.176	**< 0.001**	**< 0.001**
		Temporal	20.00 ± 0.82	19.72 ± 1.19	19.15 ± 1.97	**0.037**	1.000	**0.038**	0.256
PD		6 mm Full area	0.47 ± 0.01	0.47 ± 0.02	0.45 ± 0.03	**0.001**	0.210	**< 0.001**	**< 0.001**
	Inner ring	Average	0.46 ± 0.03	0.48 ± 0.02	0.48 ± 0.03	**0.029**	0.174	**0.030**	1.000
		Superior	0.47 ± 0.03	0.49 ± 0.03	0.50 ± 0.04	**0.001**	**0.042**	**0.001**	0.581
		Nasal	0.47 ± 0.05	0.49 ± 0.03	0.48 ± 0.03	0.156	0.188	0.498	1.000
		Inferior	0.48 ± 0.02	0.49 ± 0.03	0.50 ± 0.03	**0.006**	0.674	**0.005**	0.137
		Temporal	0.42 ± 0.05	0.43 ± 0.03	0.44 ± 0.07	0.097	1.000	0.098	0.582
	Outer ring	Average	0.49 ± 0.01	0.48 ± 0.02	0.45 ± 0.04	**< 0.001**	0.341	**< 0.001**	**< 0.001**
		Superior	0.50 ± 0.02	0.50 ± 0.02	0.47 ± 0.04	**< 0.001**	1.000	**< 0.001**	**< 0.001**
		Nasal	0.47 ± 0.03	0.46 ± 0.03	0.41 ± 0.05	**< 0.001**	0.427	**< 0.001**	**< 0.001**
		Inferior	0.50 ± 0.02	0.49 ± 0.03	0.45 ± 0.05	**< 0.001**	0.208	**< 0.001**	**< 0.001**
		Temporal	0.48 ± 0.02	0.48 ± 0.03	0.46 ± 0.05	**0.015**	1.000	**0.016**	0.136

Values are presented as means ± standard deviations.

PD, perfusion density; VD, vessel density.

*P-value from one-way analysis of variance.

^†^Post hoc test between the Group 1 and Group 2.

^‡^Post hoc test between the Group 1 and Group 3.

^§^Post hoc test between the Group 2 and Group 3.

Significant P-values are bolded.

When average VDs were compared across the four sectors of the inner ring, we found that average VD increased significantly with increased AL, with values of 17.83 ± 0.93, 18.24 ± 0.96, and 18.58 ± 1.17 mm-1 for Groups 1–3, respectively (P = 0.009). Similarly, the average PD in the inner ring increased significantly with increasing AL, with values of 0.46 ± 0.03, 0.48 ± 0.02, and 0.48 ± 0.03 for Groups 1–3, respectively (P = 0.029). Post hoc analysis revealed that values only differed significantly between Groups 1 and 3. In sector analysis, the superior and inferior sectors in VD and PD showed significant differences among all three groups (all P < 0.01).

In contrast to the inner ring, average VD in the outer ring decreased significantly with increasing AL, with values of 19.43 ± 0.60, 19.06 ± 0.81, and 18.00 ± 1.43 mm-1 for Groups 1–3, respectively (P < 0.001). Similarly, average PD decreased significantly with increasing AL, with values of 0.49 ± 0.01, 0.48 ± 0.02, and 0.45 ± 0.04 for Groups 1–3, respectively (P < 0.001). Post hoc analysis revealed that the VD and PD values of Group 3 were significantly lower than those of Groups 1 and 2 across all four sectors; average values also were significantly lower, except those of the temporal sector (all P < 0.05). These results did not change even after adjustment for clinical variables such as age, sex, BCVA, and IOP.

### Relationship between VD and clinical factors

**Fig 3:**
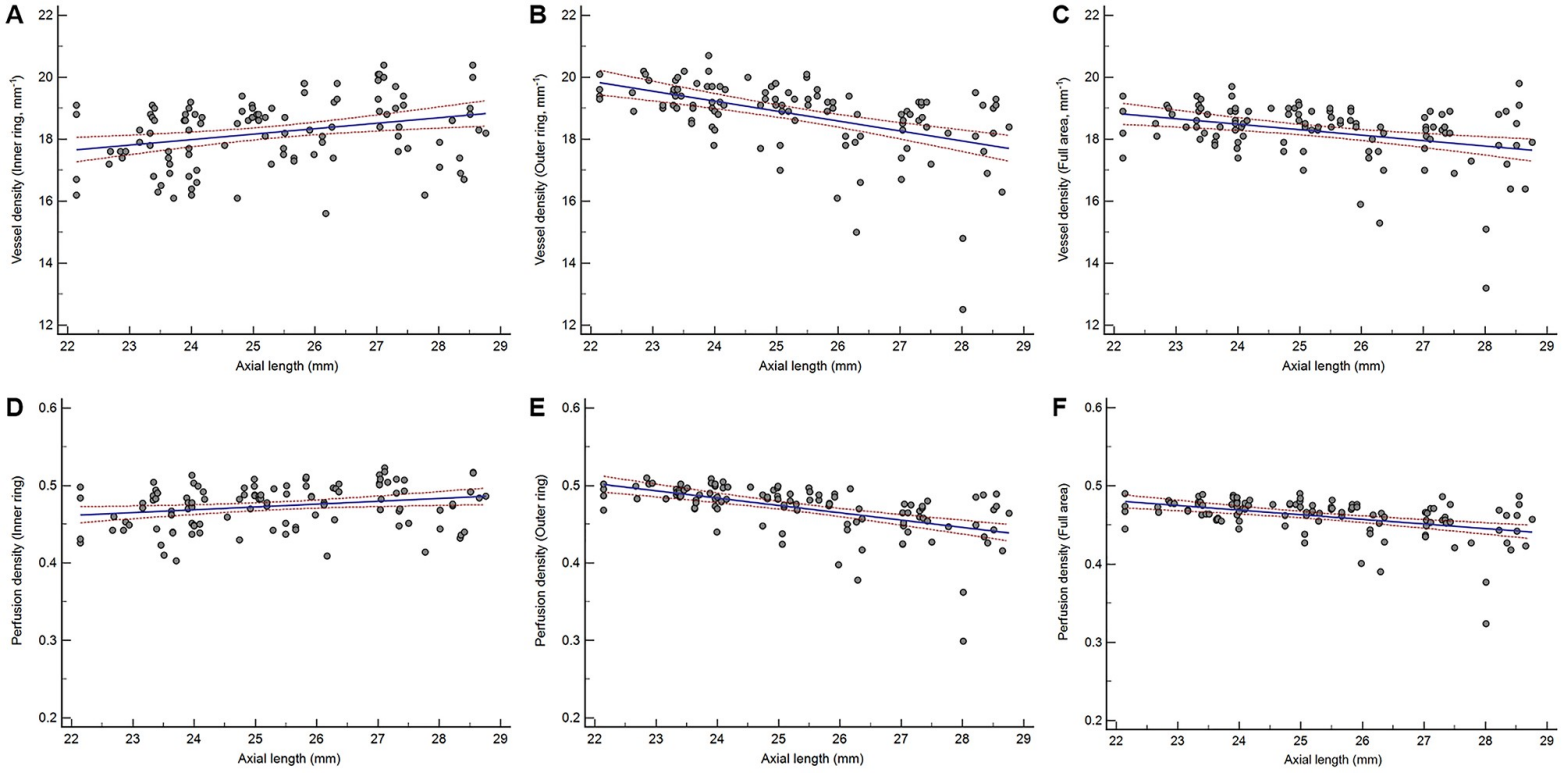
Scatter plots and estimated regression lines for axial length vs. (A) mean VD in the inner ring (r = 0.300, P = 0.001), (B) mean VD in the outer ring (r = -0.490, P < 0.001), (C) mean VD in the full area (r = -0.341, P < 0.001), (D) mean PD in the inner ring (r = 0.237, P = 0.012), (E) mean PD in the outer ring (r = -0.541, P < 0.001), and (F) mean PD in the full area (r = -0.441, P < 0.001).

Linear regression analyses were performed to examine the factors affecting VD measurements in the inner and outer ring areas. Average VD in the inner ring was significantly associated with the SE (r = –0.375; P < 0.001), AL (r = 0.300; P = 0.001), and VD of the full area (r = 0.316; P = 0.001). Average VD in the outer ring was significantly associated with the SE (r = 0.210; P = 0.027), AL (r = –0.490; P < 0.001), average RNFL thickness (r = 0.477; P < 0.001), and VD of the full area (r = 0.931, P < 0.001). Average VDs of the inner and outer rings were not significantly related (r = –0.007; P = 0.938; Table 3; Fig 3).

**Table 3:** Linear regression analysis between clinical factors and average vessel density of inner and outer ring.

	Inner ring average			Outer ring average		
	B	r	p-value	B	r	P-value
Age	-0.013 ± 0.009	-0.143	0.136	0.000 ± 0.010	0.001	0.995
Sex (female = 1)	0.043 ± 0.204	0.020	0.834	-0.290 ± 0.225	-0.123	0.200
BCVA	-0.166 ± 1.457	-0.011	0.909	2.964 ± 1.597	0.175	0.066
Spherical equivalent	-0.123 ± 0.029	-0.375	**< 0.001**	0.077 ± 0.034	0.210	**0.027**
IOP	0.023 ± 0.035	0.063	0.510	0.061 ± 0.039	0.150	0.117
Axial length	0.177 ± 0.054	0.300	**0.001**	-0.321 ± 0.055	-0.490	**< 0.001**
Average RNFL	0.002 ± 0.009	0.024	0.806	0.049 ± 0.009	0.477	**< 0.001**
Inner ring average*				-0.008 ± 0.107	-0.007	0.938
Outer ring average*	-0.007 ± 0.086	-0.007	0.938			
Full area*	0.359 ± 0.103	0.316	**0.001**	1.177 ± 0.044	0.931	**< 0.001**

Values are presented as mean ± standard deviations.

BCVA, best corrected visual acuity; IOP, intraocular pressure; RNFL, retinal nerve fiber layer.

Significant P-values are bolded.

### Artifacts of OCTA images according to axial length

We also measured the lack of vascular detection area around the optic disc, defined as “Black area (Fig 2)”. The black areas showed significant negative correlation (r = -0.705, p<0.001) with average VD in the inner ring and was decreased as the AL increased (Group 1: 0.44 ± 0.27 mm^2^, Group 2: 0.32 ± 0.25 mm^2^, Group 3: 0.25 ± 0.23 mm^2^, p = 0.010; Table 4). Similarly, the proportion of black area in the inner ring was decreased with increased AL. (Group 1: 7.12 ± 4.37%, Group 2: 5.17 ± 4.03%, Group 3: 4.05 ± 3.75%).

**Table 4:** Comparison of the black area among the three groups.

		Group 1	Group 2	Group 3	P-value*	P-value†	P-value‡	P-value§
		(< 23.99 mm)	(24–25.99 mm)	(≥ 26 mm)				
Area (mean±SD, mm^2^)	InnerSuperior	0.08±0.07	0.05±0.07	0.02±0.04	**<0.001**	**0.035**	**<0.001**	0.158
	InnerNasal	0.07±0.11	0.03±0.06	0.02±0.05	**0.007**	**0.025**	**0.012**	1.000
	InnerInferior	0.06±0.05	0.06±0.06	0.04±0.06	0.122	1.000	0.360	0.141
	InnerTemporal	0.22±0.12	0.18±0.11	0.18±0.15	0.189	0.322	0.356	1.000
	InnerRing	0.44±0.27	0.32±0.25	0.25±0.23	**0.010**	**0.035**	**<0.001**	0.158
Proportion^Π^ (mean±SD, %)	InnerSuperior	5.36±4.53	3.09±4.28	1.25±2.65	**<0.001**	**0.035**	**<0.001**	0.158
	InnerNasal	4.66±6.86	1.78±3.62	1.18±3.03	**0.007**	**0.025**	**0.012**	1.000
	InnerInferior	3.87±3.28	4.18±4.18	2.36±3.98	0.122	1.000	0.360	0.141
	InnerTemporal	14.58±7.93	11.63±6.97	11.42±9.70	0.189	0.322	0.356	1.000
	InnerRing	7.12±4.37	5.17±4.03	4.05±3.75	**0.010**	**0.035**	**<0.001**	0.158

Π The percentage of the black area in each subfield.

*P-value from one-way analysis of variance.

^†^Post hoc test between the Group 1 and Group 2.

^‡^Post hoc test between the Group 1 and Group 3.

^§^Post hoc test between the Group 2 and Group 3.

Significant P-values are bolded.

## Discussion

Changes in VDs and RNFL thicknesses according to degree of myopia have been reported in several studies. According to Leung et al., [10] peripapillary RNFL thickness measured by OCT with a peripheral radius of 3.4 mm in the center of the optic disc is thinner in subjects with high myopia than in those with mild myopia; another study showed that longer ALs are associated with reduced macular thickness and volume [15]. In addition, OCTA observations by Al-Sheikh et al. [16] revealed that high myopia is associated with reduced foveal capillary density and increased choriocapillaris flow deficit.

In the present study, we analyzed changes in VD and PD according to AL by dividing the peripapillary microvascular region of the 6 × 6 mm area measured using OCTA into the inner ring, outer ring, and full area. With increasing AL, VD and PD in the inner ring increased significantly. Conversely, VD and PD in the outer ring and 6-mm full area decreased with increasing AL. The results of linear regression analyses revealed that inner ring VDs were positively correlated with AL, whereas outer ring VDs were negatively correlated. Inner and outer ring VDs were not related to each other. Thus, OCTA measurements in the inner and outer rings may have individual characteristics and might be affected by different factors.

The superficial RNFL surrounding the optic disc is supplied by the posterior ciliary artery and central retinal arteries. The RNFL around the optic nerve head is also supplied by radial peripapillary capillaries (RPCs). It is challenging to specifically image RPCs using fluorescein angiography. However, with the introduction of OCTA, RPCs located in the SCP level can now be visualized [17]. Moreover, since the microvascular density of SCP can be measured using OCTA, it is possible to estimate the distribution of the measured value of RPC. In this study, the VD and PD of the outer ring and full area were reduced with increasing AL, and might be affected by two factors. First, this may be related to the distribution of RPCs. Previous studies have reported that RPCs were present within 7.6 mm of the optic disc, and their volume gradually decreased with increasing distance (i.e., 0.5, 2.5, and 5 mm) from the optic disc [17]. As AL increases, the OCT focus moves forward, and more structures are included in the same scan area because of image magnification [14]. Using the same scan protocol (e.g., 3 × 3 mm or 6 × 6 mm scan), the measured area expands outward as the AL becomes longer. Therefore, the distance of the measurement area from the optic disc increases with increasing AL; conversely, the RPC density decreases in a distance-dependent manner from the optic disc. Second, as the range of OCTA measurements becomes farther from the optic disc, the running blood vessels and RNFL thickness become thinner and the distribution of blood vessels becomes smaller. As the ALs of the outer ring and full area increase, VD and PD are reduced.

In contrast to the outer ring, the inner ring tended to increase with increasing AL, which may have been influenced by the lack of vascular detection area around the optic disc (Fig 2). Generally, the optic disc is located slightly behind the retinal plane, and the large vessels of the optic disc are often undetected, appearing black in OCTA images (Fig 2A and 2B). However, in long eyes, the optic disc is tilted and large vessels in superior, inferior, and nasal areas are relatively well-detected; black areas appear only in the temporal area (Fig 2C and 2D). Therefore, AL elongation causes differences in the image artifacts of the peripapillary area, and it is thought that these differences may have affected the OCTA measurements of the inner ring. In addition, because of image magnification error induced by AL variation, with longer AL and wider measurement area, the measurement area extends to the periphery of the optic disc, resulting in a reduced proportion of the optic disc within the 3 mm inner ring. Therefore, as the RPC and retinal large vessels are gradually included in the 3 mm inner ring, the VD of the 3 mm inner ring may increase with increasing AL.

The RPC network is the most superficial part of the retinal vasculature, running parallel to nerve fibers. Thus, the RNFL is thicker in the area where the RPC network is present. Previous studies reported that RPCs in the peripapillary retina were more dense in the thick RNFL area [18]. In general, the volume of RPC is thickest on the superior temporal and inferior temporal sides, and is known to have a positive correlation with the RNFL thickness [18]. Considering the association between RPC and the RNFL, it is assumed that our results in the outer ring are probably related to the distribution of RPC reported in previous studies [18–20].

The present study identified associations of peripapillary OCTA and OCT parameters. Average RNFL thicknesses were correlated with both peripapillary VDs and PDs in the outer ring, indicating that RNFL thickness may be related to peripapillary retinal microcirculation. The average RNFL thickness around the optic disc became thinner with increasing AL, which was thought to result from the histopathological features of the eyeball. In myopic eyes, the sclera becomes thinner as the posterior region expands [21]. The reductions of RNFL thickness and peripapillary perfusion parameters can therefore be attributed to eyeball elongation. In the present study, average RNFL thickness decreased significantly with increasing AL. The reductions in VD and PD in the outer ring and full area with increasing AL may be associated with a reduction in peripapillary RNFL thickness.

This study had some limitations. First, this study was retrospectively analyzed. Second, we did not analyze the peripapillary microvasculature in the deep retinal layer. However, the superficial retinal layer can be more accurately analyzed than the deep retinal layer due to the projection of artifacts in OCTA [22]. Third, the black areas were significantly associated with the OCTA measurements in the inner ring, but we could not exclude these areas because image modification was not available in the Angioplex software. Fourth, we did not exclude the large vessels in the vasculature analysis. Unlike PD, in the process of calculating the VD, perfused vessels are converted into lines, so large vessels and small vessels have the same weight. Although it is believed that large vessels might not significantly affect the analysis process, there would be some proportion of large vessels in the VDs and PDs. Finally, it is assumed that VDs and PDs in the inner and outer ring would be related to ocular magnification [23], the adjusted scan area according to magnification was not applied in the analysis. If the AL is longer than the model eye of the device, 6mm diameter scan circle will cover a wider area than actual 6x6mm retina, so the area to be analyzed should be smaller than 6x6mm circle. On the contrary, in the case of a short eye, since the adjusted circle size becomes larger than 6mm, the area that needs to be analyzed exceeds the 6x6mm scan image. The modification of the scan circle might not be applied in most of Group A. Considering the tendency of VDs and PDs in the three groups, the AL affects OCTA measurements significantly. However, we did not reveal how much magnification contributes. Further research using scan size modification will be needed. The strengths of this study are as follows: this is the first study to analyze the peripapillary region up to an area of 6 x 6 mm, and to analyze the effect of AL on the density of microvasculature in the inner and outer rings according to the distance from the optic disc.

In conclusion, we found that OCTA measurements of the peripapillary microvasculature were significantly affected by AL in myopic eyes, and that AL affected the inner and outer rings differently. In the inner ring, VD and PD increased with increased AL, whereas in the outer ring, VD and PD decreased with increased AL. There was no correlation between OCTA measurements in the inner and outer ring. We presume that image artifacts in the inner ring have caused this difference. Therefore, the effects of AL on peripapillary microvasculature depend on the measurement area, and physicians should consider the effect of AL when analyzing OCTA results.

## Supporting information

S1 Data (XLSX)
